# Effects of Lidocaine-Derived Organic Compounds on Eosinophil Activation and Survival

**DOI:** 10.3390/molecules28155696

**Published:** 2023-07-27

**Authors:** Seung-Heon Shin, Mi-Kyung Ye, Mi-Hyun Chae, Sang-Yen Geum, Ahmed S. Aboraia, Abu-Baker M. Abdel-Aal, Wesam S. Qayed, Hend A. A. Abd El-wahab, Ola F. Abou-Ghadir, Tarek Aboul-Fadl

**Affiliations:** 1 Department of Otolaryngology-Head and Neck Surgery, School of Medicine, Daegu Catholic University, Daegu 42472, Republic of Korea; miky@cu.ac.kr (M.-K.Y.); leonen@hanmail.net (M.-H.C.); saye60@naver.com (S.-Y.G.); 2 Department of Medicinal Chemistry, Faculty of Pharmacy, Assiut University, Assiut 71526, Egypt; ahmed.mohamed15@pharm.aun.edu.eg (A.S.A.); wesam.qayed@aun.edu.eg (W.S.Q.); hendaboelmaged@aun.edu.eg (H.A.A.A.E.-w.); fadl@aun.edu.eg (T.A.-F.); 3 Department of Pharmaceutical Organic Chemistry, Faculty of Pharmacy, Assiut University, Assiut 71526, Egypt; abubaker.elsayed@pharm.aun.edu.eg (A.-B.M.A.-A.); olaghadir@aun.edu.eg (O.F.A.-G.)

**Keywords:** lidocaine analog, anti-inflammation, pharmacophore, ligand-based drug

## Abstract

Lidocaine, a local anesthetic, is known to possess anti-inflammatory properties. However, its clinical use is limited by inconveniences, such as its local synesthetic effects. This study evaluated lidocaine analogs designed and synthesized to overcome the disadvantages of lidocaine, having anti-inflammatory properties. Interleukin 5 (IL-5)-induced eosinophil activation and survival were evaluated using 36 lidocaine analogs with modified lidocaine structure on the aromatic or the acyl moiety or both. Eosinophil survival was evaluated using a CellTiter 96^®^ aqueous cell proliferation assay kit. Superoxide production was determined using the superoxide dismutase-inhibitable reduction of cytochrome C method. Eosinophil cationic protein (ECP), IL-8, and transcription factor expression were determined using enzyme-linked immunosorbent assay. The platelet-activating factor (PAF)-induced migration assay was performed using a Transwell insert system. Compounds EI137 and EI341 inhibited IL-5-induced eosinophil survival and superoxide and ECP production in a concentration-dependent manner. These compounds also significantly reduced IL-8 production. Although compounds EI137 and EI341 significantly reduced phosphorylated ERK 1/2 expression, they did not influence other total and phosphorylated transcription factors. Moreover, 1000 µM of compound EI341 only inhibited PAF-induced migration of eosinophils. Lidocaine analogs EI137 and EI341 inhibited IL-5-mediated activation and survival of eosinophils. These compounds could be new therapeutic agents to treat eosinophilic inflammatory diseases.

## 1. Introduction

Eosinophils are multifunctional leukocytes that interact with various immune cells and modulate their functions. They play a role in body defense mechanisms and the development of allergic or nonallergic eosinophilic inflammatory diseases [1,2]. Tissue eosinophilia is a histological hallmark of eosinophilic chronic rhinosinusitis, allergic rhinitis, and other eosinophilic diseases. Increased eosinophil differentiation, increased adhesion and chemotaxis through the vascular endothelium, and increased survival are essential for eosinophilia. Eosinophil activation with degranulation is important for host defense against non-phagocytable pathogens and for modulating inflammatory responses [3]. The inhibition of eosinophilia and eosinophil activation are important targets in controlling eosinophil-associated diseases. Interleukins (ILs) play a dual role in eosinophilic inflammation and anti-inflammatory responses. IL-4, IL-5, and IL-13, which are produced by T-helper 2 cells, promote eosinophilic inflammation by promoting eosinophil activation, survival, and recruitment. Among them, IL-5 is a critical cytokine for eosinophil development, activation, and survival [2]. IL-10 produced by regulatory T cells is an important anti-inflammatory cytokine and can directly interact with eosinophils and inhibit their functions [4].

**Figure 1:**
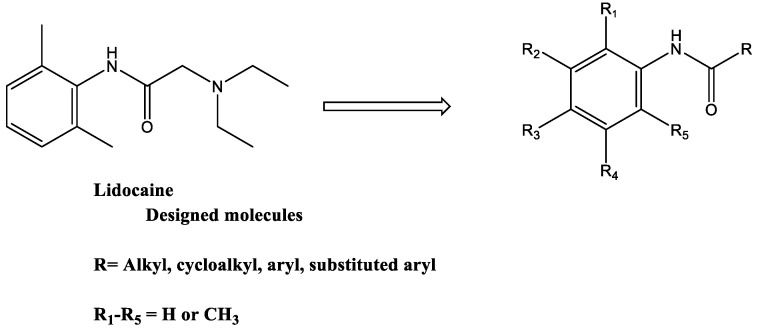
General structure of the new lidocaine analogs.

Lidocaine is commonly used as a short-acting local anesthetic agent. It also has anti-arrhythmic and anti-inflammatory effects [5,6]. Inhaled lidocaine effectively reduces the use of glucocorticoids in patients with moderate to severe asthma and has beneficial effects on bronchospasm and bronchial hyperreactivity in response to various stimuli [7,8]. Lidocaine could be an excellent candidate for replacing glucocorticoids in controlling the symptoms of asthma. However, nebulized lidocaine can cause airway irritation with bronchoconstriction, and its local airway anesthetic effects cause discomfort to the patients, indicating that lidocaine has limitations in clinical usage [7]. Therefore, several lidocaine derivatives were developed to overcome these limitations [9,10]. The developed molecules involved modifications of the aromatic ring of lidocaine, such as JMF2-1 and JM25-1, which led to antispasmodic and anti-inflammatory properties with reduced local anesthetic properties besides effects on airway hyperreactivity and bronchoconstriction [9,10]. Other modifications involved changes in the acyl moiety of the lidocaine, which resulted in an improvement of the in vitro anti-eosinophilic activities; however, they were not subject to in vivo investigations [11,12]. In spite of the variable studies on the lidocaine-derived analogs conducted, none of these analogs are used clinically. The current study developed new lidocaine analogs with modifications on both the aromatic and the acyl moieties based on a built pharmacophore generated from a training set of 16 reported compounds. The design of these molecules was based on the hypothesis that the beneficial effects of lidocaine in the treatment of asthma due to significant effects on eosinophils are not attributed to sodium-channel-blocking activity [11,12]. Accordingly, a modified lidocaine structure lacking the tertiary amine responsible for sodium-channel-blocking activity with the following general structure was designed and synthesized (Figure 1).

The objective of this study is to investigate the potential inhibitory effects of lidocaine-derived organic compounds on IL-5-mediated activation and survival of eosinophils, as well as to explore their immunomodulatory properties.

## 2. Results

### 2.1. Pharmacophore-Based Design of Lidocaine Modified Analogs

**Figure 2:**
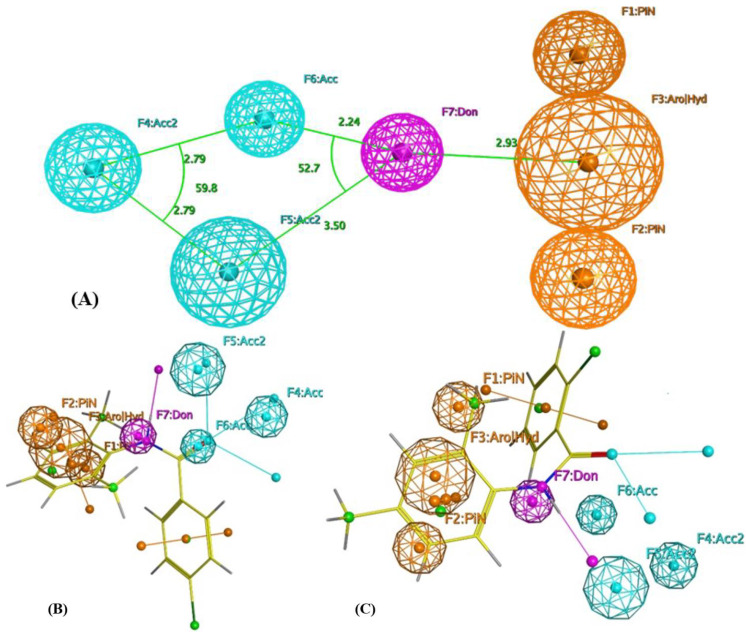
(**A**) Pharmacophoric features of eosinophil inhibitors. Acc: Hydrogen bond acceptor; Don: Hydrogen bond donor; Aro׀Hyd: Aromatic or hydrophobic; PiN: Aromatic or Pi-regular ring. (**B**) Alignment of compound EI137 to the built pharmacophore. (**C**) Alignment of compound EI341 to the built pharmacophore.

The produced pharmacophore is composed of seven features: three hydrogen bond acceptor features (Acc); one hydrogen bond donor feature (Don); one aromatic or hydrophobic (Aro׀Hyd); two aromatic or Pi-regular rings (PiN). The interfeature distance ranges between 2.24 and 3.50 Ǻ (Figure 2A). For the internal validation, the model was able to identify all the active from inactive compounds in the training set. This step assures the model’s selectivity. Based on the generated pharmacophore, the designed molecules considered modifications of lidocaine structure lacking the tertiary amine responsible for sodium-channel-blocking activity with the general structure shown in Figure 1. The pharmacophore also shows the acceptance of projection of the initial structure around the amide aromatic constituent with more hydrophobic projection and the availability of extension of the acyl part with more hydrogen acceptor features.

Alignment of the proposed compounds to the produced pharmacophore showed good hits, as can be seen from Figure 2b,c. The selected compounds for synthesis were allowed to produce either a complete or partial hit to the built pharmacophore with the condition of considering F1–F3 and F7 as essential features, as they were the most common features in the training set.

**Figure 3:**
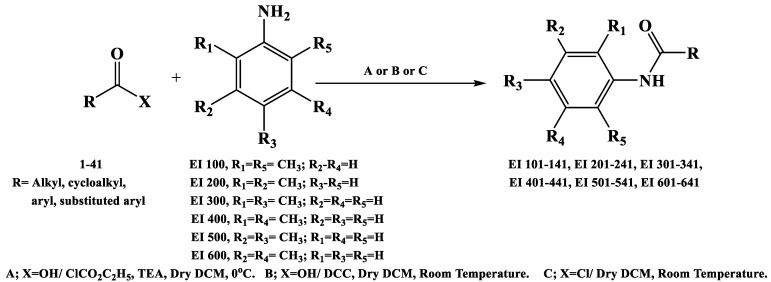
Synthesis of the target molecules.

Accordingly, a library of 36 organic molecules (EI137–EI641) was obtained through classical amidation methods. Appropriate dimethylaniline was reacted with the suitable carboxylic acid chloride, carboxylic acid anhydride, or mixed carboxylic–carbonic anhydrides. The latter was obtained in situ as an intermediate compound from the reaction of the appropriate carboxylic acid with ethyl chloroformate at 0 °C in DCM [13]. The structures of the produced compounds were confirmed by spectral methods of analysis—IR, 1 HNMR, and mass—and were consistent with the proposed ones. Their purity was confirmed with elemental analysis. Figure 3 and Table S1 present an overview of a reaction to obtain the target compounds and their structures. Yields, melting points, and spectral data are given in the Supplementary Materials (Table S2).

### 2.2. Effects of the Lidocaine Analogs on the Survival of Eosinophils

**Figure 4:**
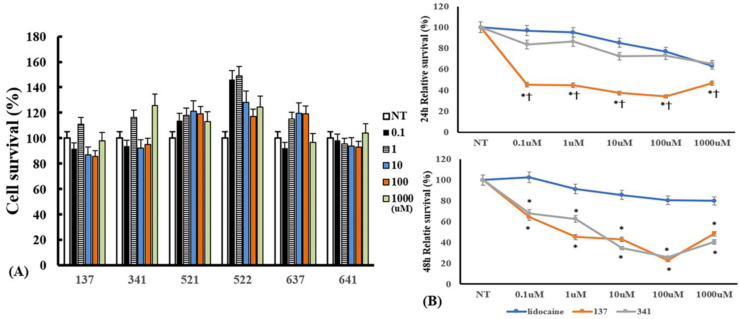
The cytotoxic effect of lidocaine analogs on eosinophils and their effects on IL-5-induced eosinophil survival: (**A**) 0.1–1000 µM of the compounds did not have cytotoxic effects on eosinophils; (**B**) pre-treatment with compounds EI137 and EI341 suppressed IL-5-induced eosinophil survival. At 24 h, compound EI137 exhibited a stronger reduction in IL-5-induced eosinophil survival compared to both lidocaine and compound EI341 at a concentration of 0.1 µM or more. At 48 h, compounds EI137 and EI341 strongly reduced IL-5-induced eosinophil survival at a concentration of 0.1 µM or more. NT, not-treated; * *p* < 0.05 compared with lidocaine; † *p* < 0.05 compared with compound EI341, *n* = 5.

To determine the optimal concentration of the organic compounds, we determined the cytotoxic effects of the organic compounds on eosinophils. Eosinophils were incubated with 0.1–1000 μM of the compounds for 24 h. The concentration of organic compounds EI137, EI341, EI521, EI522, EI637, and EI641 up to 1000 μM did not influence the viability of eosinophils (Figure 4). When the eosinophils were pre-treated with compounds for 1 h and then cultured with 50 pg/mL of IL-5 for 48 h, compound EI137 reduced IL-5-induced eosinophil survival 24 and 48 h after treatment, and compound EI341 reduced eosinophil survival at 48 h. IL-5-induced eosinophil survival was more strongly reduced by compound EI137 than by compound EI347 and lidocaine at 24 h. After 48 h, compounds EI137 and EI341 strongly reduced eosinophil survival compared with lidocaine (Figure 4).

### 2.3. Effects of the Lidocaine Analogs on Superoxide Production from Eosinophils

**Figure 5:**
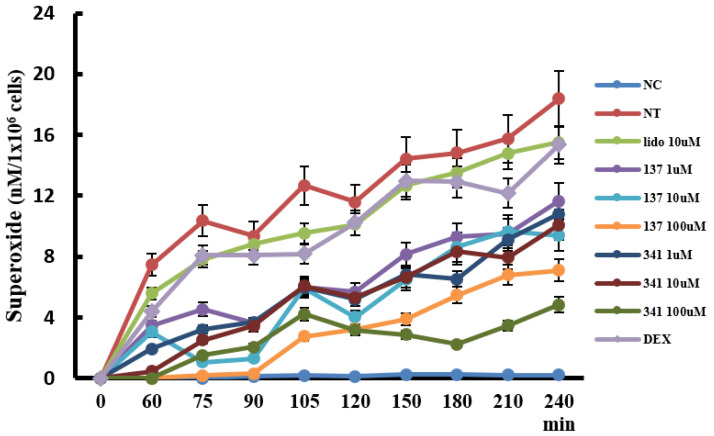
The effects of lidocaine analogs on the production of superoxide from eosinophils. Kinetics of eosinophil superoxide production; the production of superoxide increased in a time-dependent manner, and compounds EI137 and EI341 reduced IL-5-induced superoxide production. NC, negative control; NT, not-treated; Lido, lidocaine; Dex, dexamethasone; *p* < 0.05 compared with the NT group; *p* < 0.05 compared with the Lido and Dex groups, *n* = 5.

IL-5 strongly affects the development, survival, and activation of eosinophils. To determine the appropriate organic compounds for further studies, we pre-treated eosinophils with 36 organic compounds (Table S1) for 1 h and then treated them with 25 ng/mL of IL-5 to induce superoxide production from eosinophils for 4 h. Among the tested molecules, compounds EI137 and EI341 significantly inhibited superoxide production from eosinophils. These superoxide production inhibitory effects were even stronger than those of lidocaine and dexamethasone (Figure 5). Compounds EI521, EI522, EI637, and EI641 also tended to influence the production of superoxide, but not in a time- and concentration-dependent manner. Other compounds did not influence the production of superoxide. Then, we further studied compounds EI137 and EI341.

### 2.4. Effects of the Synthesized Compounds on Eosinophil Cationic Protein (ECP) and IL-8 Production from Eosinophils

**Figure 6:**
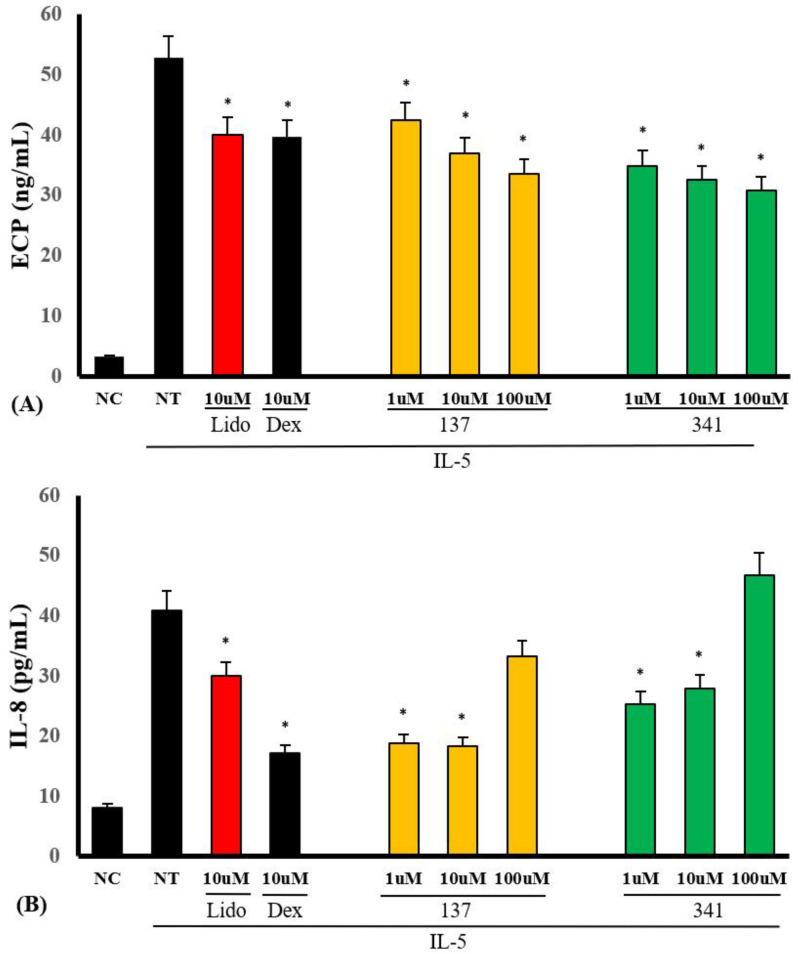
The effects of lidocaine-derived compounds EI137 and EI341 on IL-5-induced ECP and interleukin (IL)-8 production from eosinophils. (**A**) IL-5-induced ECP production was significantly inhibited by compounds EI137 and EI341 in a concentration-dependent manner. (**B**) IL-5-induced IL-8 production was significantly inhibited by the compounds at doses of 1 and 10 µM. NC, negative control; NT, not-treated; Lido, lidocaine; Dex, dexamethasone; * *p* < 0.05 compared with the NT group, *n* = 5.

We examined whether organic compounds EI137 and EI341 can inhibit IL-5-induced ECP and IL-8 production. Eosinophils were pre-treated with serial dilutions of organic compounds, from 1 to 100 μM, and then treated with 25 ng/mL of IL-5 for 4 h. IL-5-induced ECP production was significantly inhibited by compounds EI137 and EI341 in a concentration-dependent manner. IL-5-induced IL-8 production was inhibited by 1 and 10 μM of compounds EI137 and EI341 (Figure 6). However, compounds EI521, EI522, EI637, and EI641 affected ECP and IL-8 production in varied ways (Figure S1).

### 2.5. Effects of the Synthesized Compounds on Transcription Factor Expression in Eosinophils

**Figure 7:**
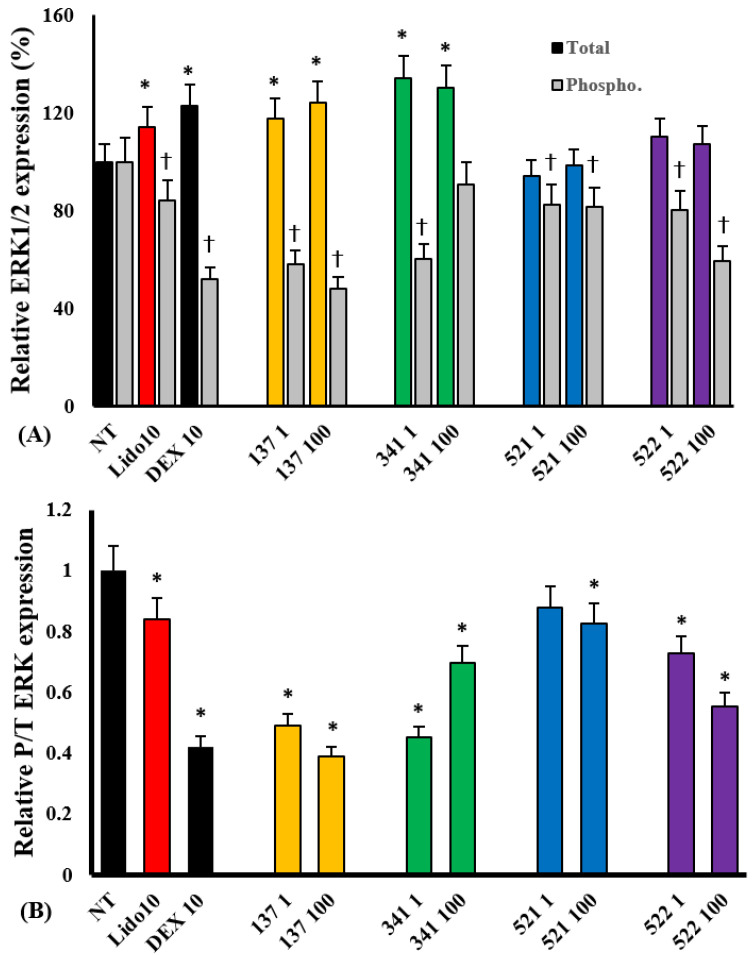
The effects of lidocaine analogs on IL-5-induced total and phosphorylated ERK 1/2 expression in eosinophils. The compounds significantly inhibited IL-5-induced phosphorylated ERK 1/2 expression (**A**) and relative phosphorylated/total ERK 1/2 expression in eosinophils (**B**); * *p* < 0.05 compared with the NT group, *n* = 7. † *p* < 0.05 compared with the Lido and Dex groups, *n* = 5.

Eosinophils rapidly express total and phosphorylated transcription factors approximately 15 min after treatment with stimulants, which is sustained up to 30 min after treatment [14]. We measured the total and phosphorylated transcription factors after 30 min of treatment with IL-5. When eosinophils were pre-treated with compounds EI137 and EI341, the total ERK 1/2 expression was significantly increased compared with the non-treated group. Phosphorylated ERK 1/2 expression was significantly reduced not only by compounds EI137 and EI341 but also by compounds EI521 and EI522. The relative total and phosphorylated ERK 1/2 expression ratios were also significantly reduced by compounds EI137, EI341, EI521, and 522 (Figure 7). However, the expression of other total and phosphorylated transcription factors was not influenced by the organic compounds (Figure S2).

### 2.6. Effects of the Synthesized Compounds on the Migration of Eosinophils by Platelet-Activating Factor (PAF)

**Figure 8:**
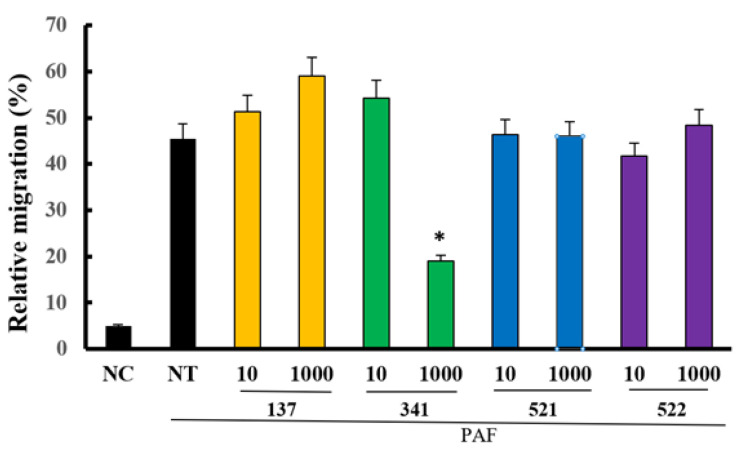
The effects of lidocaine analogs on PAF-induced eosinophil migration. Only at a dose of 1000 µM, compound EI341 significantly inhibited PAF-induced eosinophil migration. Unit = µM; NC, negative control; NT, not-treated; * *p* < 0.05 compared with the NT group, *n* = 7.

PAF is a potent inflammatory mediator that induces the chemotaxis of neutrophils and eosinophils [15]. When eosinophils were treated with 1 μM of PAF for 4 h, 45.5% ± 18.3% of the cells migrated. Furthermore, these migrations were only significantly inhibited by 1000 μM of compound EI341 (19.0% ± 7.2%). The other organic compounds did not influence the PAF-induced eosinophil migration (Figure 8).

## 3. Discussion

Lidocaine has local anesthetic and anti-inflammatory properties by inhibiting inflammatory cell or airway epithelial cell activation and cytokine production [8,16,17]. Lidocaine and its derivatives are known as potential drugs that can replace inhaled corticosteroid treatment in asthmatics [8]. Nebulized lidocaine can prevent mucus production, peribronchial fibrosis, and eosinophilic inflammation in asthma [8]. However, lidocaine has limitations in clinical usage due to its local anesthetic effect and hyperstimulation of the airway mucosa. To overcome these limitations, various studies have been conducted involving lidocaine-derived compounds that do not have these disadvantages [10,18]. In this study, we screened the 36 most active synthesized compounds, and 2 of them (EI137 and EI341) have similar anti-eosinophilic properties to lidocaine or glucocorticosteriods. Compounds EI137 and EI341 reduced IL-5-induced eosinophil activation and survival by inhibiting the ERK 1/2 pathway. However, only compound EI341 inhibited PAF-induced eosinophil migration. IL-5 time-dependently induced superoxide production and lidocaine (10 μM) reduced superoxide production, as reported previously [19]. After 4 h of treatment, compounds EI137 and EI341 strongly reduced IL-5-induced superoxide production (Figure 5). However, the other synthesized compounds did not influence or even enhance the production of superoxide from eosinophils. Therefore, we used compounds EI137 and EI341 in further studies. These compounds also significantly inhibited IL-5-induced ECP and IL-8 production from eosinophils (Figure 6). ECP and IL-8 are crucial chemical mediators that provide valuable insights into eosinophil activity and inflammatory responses. This indicates that EI137 and EI341 can effectively inhibit IL-5-induced eosinophil activation and inflammatory responses. However, it should be noted that at a higher concentration (100 µM), there was an enhancement in IL-8 production. For clinical use, it will be important to determine the appropriate concentration of these organic compounds. The produced biological data coincide with the built pharmacophore prediction, as compounds EI137 and EI341 showed good alignment with the built pharmacophore (Figure S3). Compounds EI137 and EI341 also dose-dependently inhibited mast cell degranulation, and significantly reduced the release of β-hexaminidase from RBL2H3 cells at a dose of 1000 μM (Figure S4).

IL-5 is a key mediator associated with eosinophilic inflammation and enhanced pro-duction, activation, and prolonged survival of eosinophils. IL-5 activates the Janus kinase 2, Lyn, and Raf-1, and phosphatase SHP2 pathways, which have been associated with the activation, proliferation, and survival of eosinophils [20,21,22]. IL-5 also induces the phosphorylation of ERK, MAPK, JNK, and activating transcription factor 2 [23]. Although lidocaine inhibits IL-5-induced survival and superoxide prediction, it is not involved in the phosphorylation of protein tyrosine kinase or other transcription factors [19]. Lidocaine analog compounds EI137 and EI341 significantly inhibited the expression of phosphorylated ERK1/2 but not total ERK1/2 (Figure 7). ERK1/2 functions as a protein kinase within the MAPK signaling pathway and plays a significant role in eosinophils. It promotes the survival and activation of eosinophils and regulates the production of inflammatory mediators [24,25]. The effects of EI137 and EI341 on phosphorylated ERK1/2 suggest that these compounds could suppress eosinophil survival and inflammatory responses. Although compounds EI521 and EI522 did not show an inhibitory effect on IL-5-induced activation and survival of eosinophils, they tended to inhibit the expression of phosphorylated ERK1/2. However, these organic compounds did not influence the phosphorylation of NF-κB, IκB, p38, and JNK in eosinophils. Modified lidocaine analogs can also inhibit the activation and survival of eosinophils through multiple intracellular events; for instance, they selectively interact with voltage-dependent Na^+^ channels and inhibit the flow of Na^+^ or Ca^2+^ channels with increased intracellular calcium levels [10,26]. However, the electrophysiological effects of these synthesized compounds were not assessed in this study.

Increased recruitment and survival of eosinophils are important steps in eosinophilic inflammation. Lidocaine and its modified analogs, such as tetracaine, proparacaine, and other derivatives, strongly influence eosinophilic inflammation [19,27,28]. In this study, lidocaine concentration-dependently inhibited IL-5-induced eosinophil survival (Figure 4B). At 24 h, eosinophil survival was strongly inhibited by pre-treatment of eosinophils with compound EI137 compared with that after pre-treatment with lidocaine and compound EI341. However, at 48 h, eosinophil survival was significantly inhibited by both compounds EI137 and EI341 compared to that after pre-treatment with lidocaine. Although lidocaine concentration-dependently inhibited IL-5-induced eosinophil survival, compounds EI137 and EI341 also significantly inhibited eosinophil survival from the lowest concentration (0.1 μM) to 100 μM. After that, the survival inhibitory potency was decreased. The higher concentrations of the synthesized compounds at 1000 μM may stimulate or enhance the survival of eosinophils. The lipid mediator, PAF, induces eosinophil migration from the bloodstream into tissues. Although lidocaine-derived compounds did not have as strong of an inhibitory effect on eosinophil activation or survival, compound EI341, at 1000 μM, significantly inhibited PAF-induced eosinophil migration (Figure 8). Nebulized lidocaine in human and murine models inhibits the accumulation of eosinophils in bronchial lavage fluid and airway mucosa [6,8]. Although lidocaine may not have a direct effect on eosinophil migration under in vitro conditions, lidocaine or the synthesized compounds can directly or indirectly affect the migration of eosinophils and decrease the number of eosinophils in the airway mucosa.

This study has several limitations, and our results represent a preliminary investigation with a limited set of organic compounds. IL-5 is an important cytokine that affects the activation and survival of eosinophils; however, other substances, such as PAF, IgG, and phorbol 12-myristate 13-acetate, can also affect eosinophils [19]. Confirming whether the synthesized compounds have an effect on these stimulating substances other than IL-5 will be necessary. We used eosinophils isolated from peripheral blood for this study, and the synthesized compounds reduced IL-5-induced activation and survival of eosinophils. However, pre-treatment of eosinophils with compounds cannot represent a clinical situation. To confirm the anti-inflammatory effects of these compounds, in vivo studies using animal models should be conducted. The synthesized compounds could interact with not only inflammatory cells but also structural cells in the airway mucosa. Further studies may need to confirm the local anesthetic and hyperstimulatory effects of these compounds on the airway. Although our study focused on 36 lidocaine-derived organic compounds and identified 2 compounds with anti-inflammatory effects, it is important to acknowledge the possibility that other lidocaine analogs may exhibit similar or even stronger anti-inflammatory properties. Therefore, further investigations are warranted to explore the potential of additional lidocaine-derived compounds and their anti-inflammatory properties.

## 4. Materials and Methods

### 4.1. Reagents

Lidocaine-derived organic compounds (EI137–EI641) were synthesized according to the reported methods at the Department of Pharmaceutical Organic and Medicinal Chemistry, Assiut University, Assiut, Egypt [13]. Their structures and purity were verified on the bases of spectral and elemental methods of analysis. CellTiter 96^®^ Aqueous One Solution Assay kit was purchased from Promega (Madison, WI, USA). Cytochrome C and PAF were purchased from Sigma-Aldrich (St. Louis, MO, USA). Recombinant human IL-5 and IL-8 enzyme-linked immunosorbent assay (ELISA) kits were purchased from an R&D system (Minneapolis, MN, USA). Anti-CD16 antibody was purchased from Miltenyi Biotec (Sunnyvale, CA, USA). A human eosinophil cationic protein (ECP) ELISA kit was purchased from MyBioSource (San Diego, CA, USA). The P38 mitogen-activated protein kinase (MAPK) ELISA kit and Fluo-8 calcium flux assay kit were purchased from Abcam (Cambridge, UK). Human *p*-IκB-alpha and total IκB-α were purchased from RayBio (Norcross, GA, USA). Extracellular signal-regulated kinase (ERK) 1/2, c-Jun N-terminal kinase (JNK), and NF-κB InstantOne ELISA kits were purchased from Invitrogen (Carlsbad, CA, USA).

### 4.2. Building a Pharmacophore Model

A set of 16 compounds with diverse structures and a range of activity values was used to build the 3D pharmacophore model using MOE 2020.09 software (Table S3). The pharmacophore elucidator module in MOE was used to build up the pharmacophore. The model was built based on five main common structural features: PiN, Aro, Hyd, Acc, and Don. The compounds were arranged according to activity, and the highest weight was given to the most active compounds. The conformation of the compounds was adjusted to undergo a stochastic search, and the PPCH_All scheme was used. A test set of active and inactive compounds was used to validate the efficiency of the produced pharmacophore.

### 4.3. Isolation of Eosinophils

Eosinophils were isolated from peripheral blood obtained from normal healthy volunteers after they provided informed consent, which was approved by the Institutional Review Board of Daegu Catholic University Medical Center (CR-21-184). Eosinophils were purified according to a previously described method [19]. Briefly, heparinized venous blood was layered onto 1.085 g/mL of Percoll made in piperazine-N,N′-bis(2-ethanesulfonic acid) buffer (Sigma-Aldrich, St. Louis, MO, USA) and centrifuged at 1000× *g* for 30 min. The collected granulocyte pellet was mixed with anti-CD 16 antibody conjugated with magnetic particles and cells were separated using a magnetic cell separation system. After the cells were eluted with buffer, the eosinophil count and purity were determined. The purity of the eosinophils determined using Randolph’s staining was >95%.

### 4.4. Cytotoxic Effect of Lidocaine-Derived Compounds on Eosinophils

The cytotoxic effect of the lidocaine analogs was evaluated using a CellTiter 96^®^ aqueous cell proliferation assay kit and trypan blue cell viability assay. For the cell proliferation assay, the eosinophils were cultured in a 96-well culture plate with various concentrations (0.1–1000 μM) of the compounds for 24 h at 37 °C with 5% CO_2_. Tetrazolium compound and Owen’s reagent were added to each well. The reduced tetrazolium compound produced a colored formazan product and the amount of formazan was directly proportional to the number of viable cells. Color intensities were evaluated using a fluorescence microplate reader at a wavelength of 490 nm.

### 4.5. Survival Analysis on Eosinophils

The eosinophil survival assay was performed using a CellTiter 96^®^ aqueous cell proliferation assay kit (Promega) and trypan blue cell viability assay. Eosinophils were pre-treated with various concentrations (0.1–1000 μM) of the organic compounds for 1 h and then treated with IL-5 for 48 h at 37 °C with 5% CO_2_. For the trypan blue cell viability assay, the cell suspension was stained with 0.4% trypan blue solution for 5 min at room temperature. Viable cells in the hemocytometer were counted using a microscope at ×100 magnification. The survival of eosinophils was expressed in percentages (%) relative to the non-treated group.

### 4.6. Superoxide Anion Production from Eosinophils

The generation of superoxide by eosinophils was measured by the superoxide dismutase-inhibitable reduction of cytochrome C, as described previously [19]. Freshly isolated eosinophils were suspended in Hank’s balanced salt solution (10 mM/L), N-2-hydroxyethylpiperazine-N-2′-ethanesulfonic acid buffer, 0.01% gelatin, and cytochrome C (10 μM/L) at 5 × 10^5^ cells/mL. These cells were pre-treated with various concentrations of the compounds (0.1–100 μM) and 10 μM of lidocaine or dexamethasone for 30 min, and then treated with 25 ng/mL of IL-5. Immediately after adding IL-5, the reaction wells were measured for absorbance at 550 nm using a microtiter plate autoreader (Thermoman, Molecular Devices, Menlo Park, CA, USA), followed by repeated readings at 37 °C in an incubator for 4 h. Each reaction was performed in duplicate from five samples. Superoxide anion generation was calculated using an extinction coefficient of 21.1 cm^−1^ mmol-1/L for reduced cytochrome C at 550 nm and was expressed as nanomoles of superoxide production/1 × 10^5^ cells.

### 4.7. Production of ECP and IL-8 from Eosinophils

After 4 h of treatment with IL-5, the supernatants were collected to determine the concentrations of ECP and IL-8. The levels of ECP and IL-8 in the cell-free supernatants were measured using an ELISA kit; the threshold sensitivity was 0.39 pg/mL for ECP and 2 pg/mL for IL-8.

### 4.8. Expression of Transcription Factors from Eosinophils

The total and phosphorylated NF-κB, IκB, p38, ERK1/2, and JNK were quantified using an ELISA kit according to the manufacturer’s protocol. Eosinophils were pre-treated with 1 and 100 μM of the organic compounds and treated with IL-5 for 30 min. The cells were treated with Cell Lysis Mix (combination of cell lysis buffer and enhanced solution) at room temperature for 10 min to lyse eosinophils. Then, 50 µL of the sample and 50 µL of an antibody cocktail (capture and detection antibody reagent) were added to 96-well plates at room temperature for 1 h. After adding 100 µL of detection reagent for up to 30 min, the colorimetric reaction was stopped. Transcription factor concentration was measured by reading the absorbance on a spectrophotometer at 450 nm with a reference wavelength of 655 nm.

### 4.9. Migration Assay

PAF was used as a chemoattractant for eosinophils. A migration assay was performed using a 0.3 µm pore size 24-well Transwell insert system (Corning Costar, Cambridge, MA, USA) [29]. Then, 100 µL of the eosinophils (1 × 10^6^/mL) was added to the upper chamber, and 1 µM of PAF was added to the lower chamber. After 4 h of incubation, the cells that migrated to the lower chamber were collected and counted using light microscopy.

### 4.10. Statistical Analysis

All the experiments were performed at least five times, and they showed comparable results from one experiment performed in duplicate. The results are presented as the mean ± standard deviation. Analysis of variance was performed, followed by Dunnett’s test for multiple comparisons, to determine the overall effect of pre-treatment with the organic compounds on eosinophils. A single-factor repeated-measures analysis was performed to determine the survival assay and IL-8 and ECP production (Statistical Package for the Social Sciences, version 25.0; IBM Corp., Armonk, NY, USA). Differences with *p*-value ≤ 0.05 were considered statistically significant.

## 5. Conclusions

We evaluated the effects of lidocaine-derived organic compounds on IL-5-mediated eosinophil activation, survival, and transcription factor expression. Among the synthesized compounds, EI137 and EI341 demonstrated significant anti-eosinophilic properties, including the inhibition of IL-5-induced eosinophil survival and activation through the ERK1/2 pathway. These compounds also showed inhibitory effects on IL-5-induced ECP and IL-8 production. Compound EI134 additionally exhibited significant inhibition of PAF-induced eosinophil migration. Our findings suggest that these organic compounds have potential as anti-inflammatory agents for the treatment of eosinophilic inflammatory disorders. Further studies are needed to explore their mechanisms of action and evaluate their efficacy in in vivo models and clinical settings.

## Data Availability

Data are contained within the article and Supplementary Materials.

## Supplementary Materials

The following supporting information can be downloaded at: https://www.mdpi.com/article/10.3390/molecules28155696/s1, Table S1: Structures of synthesized modified lidocaine compounds; Table S2: Yields, melting points, and spectral data of the synthesized compounds; Table S3: Training set for pharmacophore building; Figure S1: The effects of lidocaine-derived compounds EI521, E522, E637, and EI641 on IL-5-induced ECP and interleukin (IL)-8 production from eosinophils. (A) IL-5-induced ECP production was significantly inhibited by compounds EI521 and EI522. (B) IL-5-induced IL-8 production was not significantly inhibited by the compounds. Lido, lidocaine; DEX, dexamethasone; * *p* < 0.05 compared with the IL-5 group, *n* = 5; Figure S2: The effects of lidocaine analogs on IL-5-induced total and phosphorylated NF-κB expression in eosinophils. The compounds did not influence IL-5-induced phosphorylated NF-κB expression and relative phosphorylated/total NF-κB expression in eosinophils. NT, not-treated; Lido, lidocaine; DEX, dexamethasone, group, *n* = 7; Figure S3: The effects of lidocaine-derived organic compounds on RBL-2H3 cell survival and degranulation. Organic compounds did not influence the survival of RBL-2H3 cells. At a dose of 1000 µM, compounds EI137 and EI341 significantly inhibited the release of β-hexosaminidase from RBL-2H3 cells. NT, not-treated; * *p* < 0.05 compared with the NT group, *n* = 7.

## Sample Availability

Not available.
