# Supplementary material for: Effects of Lidocaine-Derived Organic Compounds on Eosinophil Activation and Survival
Source: Molecules. 2023 Jul 27;28(15):5696. doi: 10.3390/molecules28155696 (PMC10420271; doi:10.3390/molecules28155696)
Supplement: Supplementary file 1 [file molecules-28-05696-s001.zip › molecules-2514475-supplementary.pdf]

Table S1. Structures of synthesized modified lidocaine compounds.

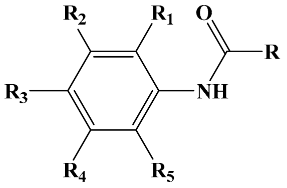

**Compdound (M.Wt.)**	**R**	**R_1**	**R_2**	**R_3**	**R_4**	**R_5**
**EI 137**	*p*-Chlorophenyl	CH_3	H	H	H	CH_3
**EI 203**	n-Hexyl	CH_3	CH_3	H	H	H
**EI 212**	Cyclopropyl	CH_3	CH_3	H	H	H
**EI 215**	Cyclohexyl	CH_3	CH_3	H	H	H
**EI 221**	Phenyl	CH_3	CH_3	H	H	H
**EI 227**	*p*-Nitrophenyl	CH_3	CH_3	H	H	H
**EI 228**	*o*-Nitrophenyl	CH_3	CH_3	H	H	H
**EI 234**	*p*-Hydroxyphenyl	CH_3	CH_3	H	H	H
**EI 301**	n-Pentyl	CH_3	H	CH_3	H	H
**EI 305**	$C_{17}H_{35}$	CH_3	H	CH_3	H	H
**EI 306**	$C_{15}H_{31}$	CH_3	H	CH_3	H	H
**EI 321**	Phenyl	CH_3	H	CH_3	H	H
**EI 327**	*p*-Nitrophenyl	CH_3	H	CH_3	H	H
**EI 328**	*o*-Nitrophenyl	CH_3	H	CH_3	H	H
**EI 337**	*p*-Chlorophenyl	CH_3	H	CH_3	H	H
**EI 341**	*o*-Chlorophenyl	CH_3	H	CH_3	H	H
**EI 401**	n-Butyl	CH_3	H	H	CH_3	H
**EI 405**	$C_{17}H_{35}$	CH_3	H	H	CH_3	
**EI 412**	Cyclopropyl	CH_3	H	H	CH_3	H
**EI 427**	*p*-Nitrophenyl	CH_3	H	H	CH_3	H
**EI 428**	*o*-Nitrophenyl	CH_3	H	H	CH_3	H
**EI 435**	*o*-Hydroxyphenyl	CH_3	H	H	CH_3	H
**EI 502**	n-Butyl	CH_3	H	H	CH_3	H
**EI 503**	n-Hexyl	H	CH_3	CH_3	H	H
**EI 505**	$C_{17}H_{35}$	H	CH_3	CH_3	H	H
**EI 506**	$C_{15}H_{31}$	H	CH_3	CH_3	H	H
**EI 521**	Phenyl	H	CH_3	CH_3	H	H
**EI 522**	2-Methylpropyl	H	CH_3	CH_3	H	H
**EI 528**	*o*-Nitrophenyl	H	CH_3	CH_3	H	H
**EI 615**	Cyclohexyl	H	CH_3	CH_3	H	H
**EI 627**	*p*-Nitrophenyl	H	CH_3	H	CH_3	H
**EI 628**	*o*-Nitrophenyl	H	CH_3	H	CH_3	H
**EI 636**	2-Hydroxy-4-chlorophenyl	H	CH_3	H	CH_3	H
**EI 637**	*p*-Chlorophenyl	H	CH_3	H	CH_3	H
**EI 641**	*o*-Chlorophenyl	H	CH_3	H	CH_3	H

Table S2. Yields, melting points, and spectral data of the synthesized compounds.

4-Chloro-N-(2,6-dimethylphenyl)benzamide (EI137)

Yield : 20 %

MP: 175-177°C (Reported174-176 °C)

^{1}H NMR (90 MHz, $CDCl_3$, δ ppm): 2.35 (s, 6H, $(C\underline{H}_3)_2\ C_6H_3$), 6.80-7.85 (m, 8H, $C_6\underline{H}_3$, $C_6\underline{H}_4$ & $N\underline{H}$).

N-(2,3-dimethylphenyl)heptanamide (EI203)

Yield: 55%;

MP: 86.5 – 87°C

^{13}C NMR (100 MHz, DMSO, δ ppm): 14.40 ($\underline{C}H_3$-CH_2-), 20.63, 22.51, 25.80, 28.82 (CH_3-$(\underline{C}H_2)_4$-), 36.17 (CO-$\underline{C}H_2$-), 124.18, 125.54, 127.22, 131.64, 136.73, 137.28 (Aryl Carbons), 171.65 ($\underline{C}$O-NH).

CHN analysis: Found/Calcd: C, 77.40; H, 10.14; N, 5.87 (C, 77.21; H, 9.93; N, 6.00)

N-(2,3-dimethylphenyl)cyclopropanecarboxamide (EI212)

Yield: 19%;

MP: 138 - 140°C;

^{1}H NMR (90 MHz, $CDCl_3$, δ ppm): 0.60-0.90 (m, 2H, $C\underline{H}HC\underline{H}HCHCO$), 0.90-1.30 (m, 2H, $CH\underline{H}CH\underline{H}CHCO$), 1.30-1.80 (m, 1H, $COC\underline{H}$), 2.10 (s, 3H, 2-$C\underline{H}_3C_6H_3$), 2.25 (s, 3H, 3-$C\underline{H}_3C_6H_3$), 6.70-7.50 (m, 4H, $C_6\underline{H}_3$ & $N\underline{H}$).

^{13}C NMR (100 MHz, DMSO, δ ppm): 14.35, 14.47 ($(\underline{C}H_2)_2$), 20.65, (CO-$\underline{C}H_2$-), 123.91, 125.55, 127.12, 131.34, 136.74, 137.29 (Aryl Carbons), 172.07 ($\underline{C}$O-NH).

CHN analysis: C, 76.48; H, 8.15; N, 7.65 (C, 76.16; H, 7.99; N, 7.40)

N-(2,3-dimethylphenyl)cyclohexanecarboxamide (EI215)

Yield: 40 %

MP: 161 – 164°C (Reported 162-163°C)

^{1}H NMR (60 MHz, $CDCl_3$, δ ppm): 1.06-2.20 (m, 10H, $(C\underline{H}_2)_5$), 2.26 (s, 3H, 2-$C\underline{H}_3C_6H_3$), 2.43 (s, 3H, 3-$C\underline{H}_3C_6H_3$), 2.30-2.53 (m, 1H, $COC\underline{H}$), 6.86-7.60 (m, 4H, $C_6\underline{H}_3$ & $N\underline{H}$).

N-(2,3-dimethylphenyl)benzamide (EI221)

Yield : 98 %

MP:189-191°C (CAS NO 3096-94-4)

^{1}H NMR (60 MHz, $CDCl_3$, δ ppm): 2.66 (s, 3H, 2-$C\underline{H}_3C_6H_3$), 2.80 (s, 3H, 3-$C\underline{H}_3C_6H_3$), 7.40-8.53 (m, 9H, $(CH_3)_2C_6\underline{H}_3$, $C_6\underline{H}_5$ & $N\underline{H}$).

N-(2,3-dimethylphenyl)-4-nitrobenzamide (EI227)

Yield%: 20 %

MP: 207 – 209°C CAS Registry Number 35709-76-3

^{1}H NMR (60 MHz, $CDCl_3$, δ ppm): 2.33 (s, 3H, 2-$C\underline{H}_3C_6H_3$), 2.46 (s, 3H, 3-$C\underline{H}_3C_6H_3$), 7.10-7.80 (m, 4H, $(CH_3)_2C_6\underline{H}_3$& $N\underline{H}$), 7.96-8.43 (dd, 4H, 4-$NO_2C_6\underline{H}_4$).

N-(2,3-dimethylphenyl)-2-nitrobenzamide (EI228)

Yield: 16%

MP: 178°C (Reported 177 – 179°C)

^{1}H NMR (60 MHz, $CDCl_3$, δ ppm): 2.20 (s, 3H, 2-$C\underline{H}_3C_6H_3$), 2.35 (s, 3H, 3-$C\underline{H}_3C_6H_3$), 6.90-7.70 (m, 7H, $(CH_3)_2C_6\underline{H}_3$&2-$NO_2C_6\underline{H}_4$), 7.90-8.10 (s, 1H, $N\underline{H}$).

4-Hydroxy-N-(2,3-dimethylphenyl)benzamide (EI234)

Yield: 26 %

MP: 150 – 152°C CAS Registry Number 459419-70-6

^{1}H NMR (60 MHz, $CDCl_3$, δ ppm): 2.26 (s, 3H, 2-$C\underline{H}_3C_6H_3$), 2.36 (s, 3H, 3-$C\underline{H}_3C_6H_3$), 6.90-8.10 (m, 9H, $(CH_3)_2C_6\underline{H}_3$&4-$OHC_6\underline{H}_4$, OH & NH).

^{13}C NMR (100 MHz, DMSO, δ ppm): 65.85 ($(C\underline{H}_3)_2C_6H_3$), 121.79, 125.18, 125.74, 128.09, 129.71, 132.83, 133.22, 136.61, 137.49, 153.12 (Aryl Carbons), 164.99 ($\underline{C}$O-NH).

N-(2,4-dimethylphenyl)hexanamide (EI301)

Yield: 26%

MP: 101-102°C CAS Registry Number 324067-84-7

^{1}H-NMR (400 MHz, $CDCl_3$, δ ppm): 0.82-0.85 (t, 3H, $CH_2C\underline{H}_3$), 1.27-1.89 (m, 4H, $(C\underline{H}_2)_2CH_3$), 1.63-1.66 (m, 2H, $C\underline{H}_2CH_2CO$), 2.12 (s, 3H, 2-$C\underline{H}_3C_6H_3$), 2.20 (s, 3H, 4-$C\underline{H}_3C_6H_3$), 2.26- 2.30 (t, 2H, $COC\underline{H}_2$), 6.91 (m,2H, $C_6\underline{H}_3$), 6.98 (Hump, H, N$\underline{H}$), 6.46-6.48 (m, 1H, $C_6\underline{H}_3$).

^{13}C-NMR (101 MHz, CDCl3, δ ppm):14.00, 17.80, 20.90, 22.47, 24.66, 31.49, 37.51,123.83, 127.51,129.77,131.12, 133.02, 135.00.

N-(2,4-dimethylphenyl)stearamide (EI305)

Yield: 30%;

MP: 83.5 – 85°C;

^{1}H NMR (90 MHz, $CDCl_3$, δ ppm): 0.95 (t, 3H, $CH_2C\underline{H}_3$), 1.1-1.9 (m, 30H, $(C\underline{H}_2)15CH_3$), 2.15 (s, 3H, 2-$C\underline{H}_3C_6H_3$), 2.25 (s, 3H, 4-$C\underline{H}_3C_6H_3$), 2.3 (t, 2H, $COC\underline{H}_2$), 6.80-7.20 (m, 3H, $C_6\underline{H}_3$), 7.30-7.50 (br s, 1H, N$\underline{H}$).

Elemental analyses calculated for $C_{26}H_{45}NO$ C, 80.56; H, 11.7; N, 3.61. Found C, 80.72; H 11.89; N 3.88.

N-(2,4-dimethylphenyl)palmitamide (EI306)

Yield: 28.5%;

MP: 99 – 102°C;

^{1}H NMR (90 MHz, $CDCl_3$, δ ppm): 0.85 (t, 3H, $CH_2C\underline{H}_3$), 1.10-1.90 (m, 26H, $(C\underline{H}_2)_{13}CH_3$), 2.20 (s, 3H, 2-$C\underline{H}_3C_6H_3$), 2.25 (s, 3H, 4-$C\underline{H}_3C_6H_3$), 2.30 (t, 2H, $COC\underline{H}_2$), 6.70-7.20 (m, 3H, $C_6\underline{H}_3$), 7.35-7.55 (br s, 1H, N$\underline{H}$).

Elemental analyses calculated for $C_{24}H_{41}NO$ C, 80.16; H, 11.49; N, 3.9. Found C, 80.43; H 11.65; N 4.11.

N-(2,4-dimethylphenyl)benzamide (EI321)

Yield: 98%;

MP: 186-189°C; (reported 193-194°C)

^{1}H NMR (60 MHz, $CDCl_3$, δ ppm): 2.33 (s, 3H, 2-$C\underline{H}_3C_6H_3$), 2.36 (s, 3H, 4-$C\underline{H}_3C_6H_3$), 6.86-8.10 (m, 9H, $C_6\underline{H}_5$, $C_6\underline{H}_5$, NH).

N-(2,4-dimethylphenyl)-4-nitrobenzamide, (EI327)

Yield: 28%;

MP: 166-169°C

^{1}H NMR (90 MHz, $CDCl_3$, δ ppm): 2.17 (s, 3H, 2-$C\underline{H}_3C_6H_3$), 2.25 (s, 3H, 4-$C\underline{H}_3C_6H_3$), 7.15-7.88 (m, 4H, $(CH_3)_2C_6\underline{H}_3$& NH), 7.99-8.53 (dd, 4H, 4-$NO_2C_6H_4$).

N-(2,4-dimethylphenyl)-2-nitrobenzamide, (EI328)

Yield:25 %;

MP: 161-162°C (Reported 161-163°C)
^{1}H NMR (90 MHz, $CDCl_3$, δ ppm): 2.15 (s, 3H, 2-C$\underline{H}_3C_6H_3$), 2.23 (s, 3H, 4-C$\underline{H}_3C_6H_3$), 7.10-7.90 (m, 4H, $(CH_3)_2C_6\underline{H}_3$ & N$\underline{H}$), 7.97-8.55 (dd, 4H, 4-$NO_2C_6\underline{H}_4$).

N-4-chloro-N-(2,4-dimethylphenyl)benzamide (EI337)
Yield: 20%;
MP: 167-8°C
^{1}H NMR (90 MHz, $CDCl_3$, δ ppm): 2.18 (s, 3H, 2-C$\underline{H}_3C_6H_3$), 2.27 (s, 3H, 5-C$\underline{H}_3C_6H_3$), 6.80-7.20 (m, 3H, $C_6\underline{H}_3$), 7.50-7.60 (br s, 1H, N$\underline{H}$), 7.30-7.80 (dd, 4H, C_6H_4)

N-2-chloro-N-(2,4-dimethylphenyl)benzamide (EI341)
Yield: 46.5%;
MP: 163-165°C
^{1}H NMR (60 MHz, $CDCl_3$, δ ppm): 2.20 (s, 6H, $(C\underline{H}_3)_2$, 6.90-8.00 (m, 8H, $(CH_3)_2C_6\underline{H}_3$, 2-$ClC_6\underline{H}_4$ & N$\underline{H}$).

N-(2,5-dimethylphenyl)pentanamide (EI401)
Yield: 35.0%;
MP:129.5°C;
^{1}H NMR (90 MHz, $CDCl_3$, δ ppm): 0.90 (t, 3H, $CH_2C\underline{H}_3$), 1.10-1.90 (m, 4H, $C\underline{H}_2C\underline{H}_2CH_3$), 2.18 (s, 3H, 2-C$\underline{H}_3C_6H_3$), 2.35 (s, 3H, 5-C$\underline{H}_3C_6H_3$), 2.35 (t, 2H, $COC\underline{H}_2$), 6.70-7.30 (m,3H, $C_6\underline{H}_3$), 7.40-7.60 (br s, 1H, N$\underline{H}$).

N-(2,5-dimethylphenyl)stearamide (EI405)
Yield: 16.5%;
MP: 187.5°C;
^{1}HNMR (90 MHz, $CDCl_3$, δ ppm): 0.90 (t, 3H, $CH_2C\underline{H}_3$), 1.10-2.00 (m, 30H, $(C\underline{H}_2)_{15}CH_3$), 2.20 (s, 3H, 2-C$\underline{H}_3C_6H_3$), 2.33 (s, 3H, 5-C$\underline{H}_3C_6H_3$), 2.40 (t, 2H, $COC\underline{H}_2$), 6.70-7.23 (m, 3H, $C_6\underline{H}_3$),7.43-7.66 (brs, 1H, N$\underline{H}$).

N-(2,5-dimethylphenyl)cyclopentanecarboxamide (EI412)
Yield: 25.3 %;
MP: 169–170°C;
^{1}H-NMR (400 MHz, $CDCl_3$, δ ppm): 0.856-0.865(m, 3H,$(C\underline{H}_2)_4$-cyclopentane), 1.088-1.12(m, 3H, $(C\underline{H}_2)_4$-cyclopentane), 1.55(m, 2H, $(C\underline{H}_2)_4$-cyclopentane),1.9(m, 1H, C$\underline{H}$CO), 2.26 (s, 3H, $(C\underline{H}_3)C_6H_3$), 2.32(s, 3H, $(C\underline{H}_3)C_6H_3$), 6.88-7.03 (m, 2H, ,$C_6\underline{H}_3$), 71.23((Hump,1H, N$\underline{H}$), 7.69 (m, 1H, $C_6\underline{H}_3$).
^{13}C-NMR (101 MHz, $CDCl_3$, δ ppm): 7.89,15.61,17.38, 21.11, 123.52, 125.39, 125.60, 130.19, 135.70, 136.45, 171.89.
Elemental analyses calculated for $C_{14}H_{19}NO$ C, 77.38; H, 8.81; N, 6.45. Found C 76.4 , H 8.16, N 7.68

N-(2,5-dimethylphenyl)-4-nitrobenzamide (EI427)
Yield:15 %
MP: 117-8°C
^{1}H NMR (90 MHz, $CDCl_3$, δ ppm): 2.33 (s, 3H, 2-C$\underline{H}_3C_6H_3$), 2.40 (s, 3H, 5-C$\underline{H}_3C_6H_3$), 7.18-7.92 (m, 4H, $(CH_3)_2C_6\underline{H}_3$& N$\underline{H}$), 8.10-8.73 (dd, 4H, 4-$NO_2C_6\underline{H}_4$).

N-(2,5-dimethylphenyl)-2-nitrobenzamide, (EI428)
Yield:15 %
MP: 175-6°C (Reported MP 173 – 175)

^{1}H NMR (90 MHz, $CDCl_3$, δ ppm): 2.36 (s, 3H, 2-$C\underline{H}_3C_6H_3$), 2.46 (s, 3H, 5-$C\underline{H}_3C_6\underline{H}_3$), 6.90-7.43 (m, 3H, $(CH_3)_2C_6H_3$), 7.50-7.86 (m, 4H, 2-$NO_2C_6\underline{H}_4$), 8.03-8.23 (br s, 1H, N$\underline{H}$).

N-(2,5-dimethylphenyl)-2-hydroxybenzamide (EI435)

Yield: 25 %

MP: 95°C (reported 164°C)

^{1}H NMR (90 MHz, $CDCl_3$, δ ppm): 2.26 (s, 3H, 2-$C\underline{H}_3C_6H_3$), 2.40 (s, 3H, 5-$C\underline{H}_3C_6H_3$), 6.23-6.43 (br s, 1H, $\underline{OH}$). 6.73-7.66 (m, 8H, $(CH_3)_2C_6\underline{H}_3$, 2-$OHC_6\underline{H}_4$ & N$\underline{H}$).

N-(3,4-dimethylphenyl)pentanamide (EI502)

Yield: 20%

MP: 107 -109°C (reported MP: 109.5–110°C)

^{1}H NMR (90 MHz, $CDCl_3$, δ ppm): 0.93 (t, 3H, $(CH_2C\underline{H}_3)$, 1.18-1.88 (m, 6H, $C\underline{H}_2C\underline{H}_2C\underline{H}_2CH_3$), 2.25 (s, 6H, $(C\underline{H}_3)_2C_6H_3$), 2.36 (t, 2H, $COC\underline{H}_2$), 6.79-7.28 (m, 3H, $C_6\underline{H}_3$), 7.48-7.67 (br s, 1H, N$\underline{H}$).

N-(3,4-dimethylphenyl) heptamide (EI503)

Yield: 16%

MP: 116 -119°C

^{1}H NMR (90 MHz, $CDCl_3$, δ ppm): 0.83 (t, 3H, $CH_2C\underline{H}_3$), 1.15-1.85 (m, 8H, $(C\underline{H}_2)_4CH_3$), 2.24 (s, 6H, $(C\underline{H}_3)_2C_6H_3$), 2.30 (t, 2H, $COC\underline{H}_2$), 7.20 (s,2H, o-C_6H_3), 7.25 (s,1H, p-C_6H_3), 7.27 (hump, 1H, N$\underline{H}$).

N-(3,4-dimethylphenyl) stearamide (EI505)

Yield: 63.0 %;

MP: 83.9°C

^{1}H NMR (400 MHz, $CDCl_3$, δ ppm): 0.95 (t, 3H, $(CH_2)_{16}C\underline{H}_3$), 1.32-1.34 (m, 28H, $(CH_2(C\underline{H}_2)_{14}CH_3)$, 1.67-1.76 (m, 2H, $COCH_2\underline{CH_2}$), 2.23 (d, 6H, C_6H_3-$(C\underline{H}_3)_2$), 2.32 (t, 2H, $COC\underline{H}_2$), 7.18 (s,2H, o-C_6H_3), 7.28 (s,1H, p-C_6H_3), 7.29 (hump, 1H, N$\underline{H}$).

Anal Calcd: C, 80.56; H, 11.70; N, 3.61. Found: 80.74, 11.98, 3.88

MS (ES) *m/z* 377.38[M+1]+

N-(3,4-dimethylphenyl)palmitamide (EI506)

MP: 88°C

Yield: 76 %.

^{1}H NMR (90 MHz, $CDCl_3$, δ ppm): 0.92 (t, 3H, $CH_2C\underline{H}_3$), 1.12-2.08 (m, 26H, $(C\underline{H}_2)_{13}CH_3$), 2.21 (s, 6H, $(C\underline{H}_3)_2C_6H_3$), 2.36 (t, 2H, $COC\underline{H}_2$), 6.66-7.16 (m, 3H, $C_6\underline{H}_3$), 7.33-7.60 (brs, 1H, NH).

Anal Calcd: C, 80.16; H, 11.49; N, 3.90. Found: 79.87, 11,63, 4.13

N-(3,4-dimethylphenyl)benzamide (EI521)

Yield: 98 %;

MP:187-190 °C; (CAS NO 27285-18-3)

^{1}H NMR (60 MHz, $CDCl_3$, δ ppm): 2.20 (s, 6H, $(C\underline{H}_3)_2C_6H_3$), 6.90-8.16 (m, 9H, $(CH_3)_2C_6\underline{H}_3$, $C_6\underline{H}_5$ & N$\underline{H}$).

3-Methyl-N-(3,4-dimethylphenyl)butanamide (EI522)

Yield: 76.5 %;

MP: 90-91°C; (CAS NO 349423-12-7)

^{1}H-NMR (90 MHz, $CDCl_3$, δ ppm): 0.80-1.30 (m, 1H, $C\underline{H}(CH_3)_2$), 0.96 (d, 6H, $CH(C\underline{H}_3)_2$), 2.16 (d, 2H, $COC\underline{H}_2$), 2.20 (s, 6H, $C_6H_3(C\underline{H}_3)_2$, 6.83-7.36 (m,4H, $C_6\underline{H}_3$ & $N\underline{H}$).
Anal Calcd:C, 76.06; H, 9.33; N, 6.82.Found: 76.34, 9.50, 6.96
MS (ES) *m/z* 377.38$[M+1]^+$

N-(3,4-dimethylphenyl)-2-nitrobenzamide (EI528)
Yield: 16.0 %;
MP: 153-155°C
IR (ύ cm^{-1}, KBr): 3268 (NH), 1655 (C=O);
^{1}H NMR (90 MHz, $CDCl_3$, δ ppm):2.20 (s, 6H, $C_6H_3(C\underline{H}_3)_2$), 6.80-8.00 (m, 8H, $C_6\underline{H}_3$, $C_6\underline{H}_4$ & $N\underline{H}$).

N-(3,5-dimethylphenyl)cyclohexanecarboxamide (EI615)
Yield: 30 %.
MP: 155°C; (CAS NO 315712-18-6)
^{1}H NMR (60 MHz, $CDCl_3$, δ ppm): 1.10-2.23 (m, 10H, $(CH_2)_5$), 2.36 (s, 6H, $(CH_3)_2$), 2.40-2.66 (m, 1H, COCH), 6.70-6.86 (br s, 1H, NH), 7.13-7.46 (m, 3H, C_6H_3).
Anal Calcd: C, 77.88; H, 9.15; N, 6.05, Found: 78.13, 9.29, 6.31
MS (ES) *m/z* 219.34 $[M+1]^+$

N-(3,5-dimethylphenyl)-4-nitrobenzamide (EI 627)
Yield : 22 %
MP: 175-177°C
^{1}H NMR (90 MHz, $CDCl_3$, δ ppm): 2.38 (s, 6H, $(CH_3)_2$), 7.15-7.88 (m, 4H, $(CH_3)_2C_6\underline{H}_3$& $N\underline{H}$), 7.99-8.53 (dd, 4H, 4-$NO_2C_6\underline{H}_4$).

N-(3,5-dimethylphenyl)-2-nitrobenzamide (EI 628)
Yield : 17.6%
MP: 165.5-167°C
^{1}H NMR (90 MHz, $CDCl_3$, δ ppm): 2.37 (s, 6H, $(CH_3)_2$), 6.90-7.43 (m, 3H, $(CH_3)_2C_6\underline{H}_3$),7.50-7.86 (m, 4H, 2-$NO_2C_6\underline{H}_4$), 8.06-8.28 (br s, 1H, $N\underline{H}$).

4-chloro-2-hydroxy-N-(3,5-dimethylphenyl)benzamide (EI 636)
Yield : 12%
MP: 208-210.5°C
^{1}H NMR (90 MHz, $CDCl_3$, δ ppm): 2.36 (s, 6H, $(C\underline{H}_3)_2C_6H_3$), 6.55-7.96 (m, 8H, $(CH_3)_2C_6\underline{H}_3$, 2-$OHC_6\underline{H}_4$, $O\underline{H}$ & $N\underline{H}$).

4-chloro-N-(3,5-dimethylphenyl)benzamide (EI 637)
Yield: 75 %;
MP: 144-145°C; (reported MP 130-133°C)
^{1}H NMR (60 MHz, $CDCl_3$, δ ppm): 2.33 (s, 6H, $(C\underline{H}_3)_2C_6H_3$),6.66-6.90 (br s, 1H, $N\underline{H}$), 6.93-8.33 (m,7H, $C_6\underline{H}_4$ & $C_6\underline{H}_3$).

2-chloro-N-(3,5-dimethylphenyl)benzamide (EI 641)
Yield : 17%
MP: 137-139°C

^{1}H NMR (60 MHz, $CDCl_3$, δ ppm): 2.36 (s, 6H, (C$\underline{H}_3$)$_2$ C_6H_3), 7.00-7.93 (m, 8H, $(CH_3)_2C_6\underline{H}_3$, 2-Cl$C_6\underline{H}_4$ & N$\underline{H}$).

Table S3. Training set for pharmacophore building.

Test set		IC_{50} (μM)
**compounds**	**Structure**	**Experimental**
**I**	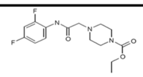	**0.18**
**II**	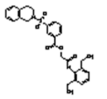	**0.133**
**III**	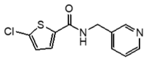	**0.257**
**IV**	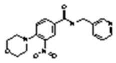	**0.257**
**V**	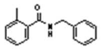	**0.019**
**VI**	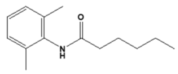	**0.062**
**VII**	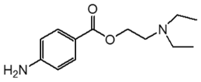	**0.071**
**VIII**	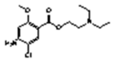	**0.25**
**IX**	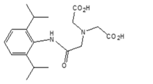	**0.161**
**X**	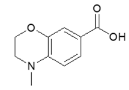	**0.092**
**XI**	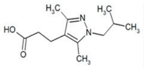	**0.015**
**XII**	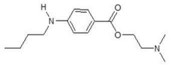	**0.222**
**XIII**	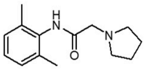	**0.106**
**XIV**	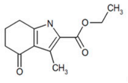	**0.19**

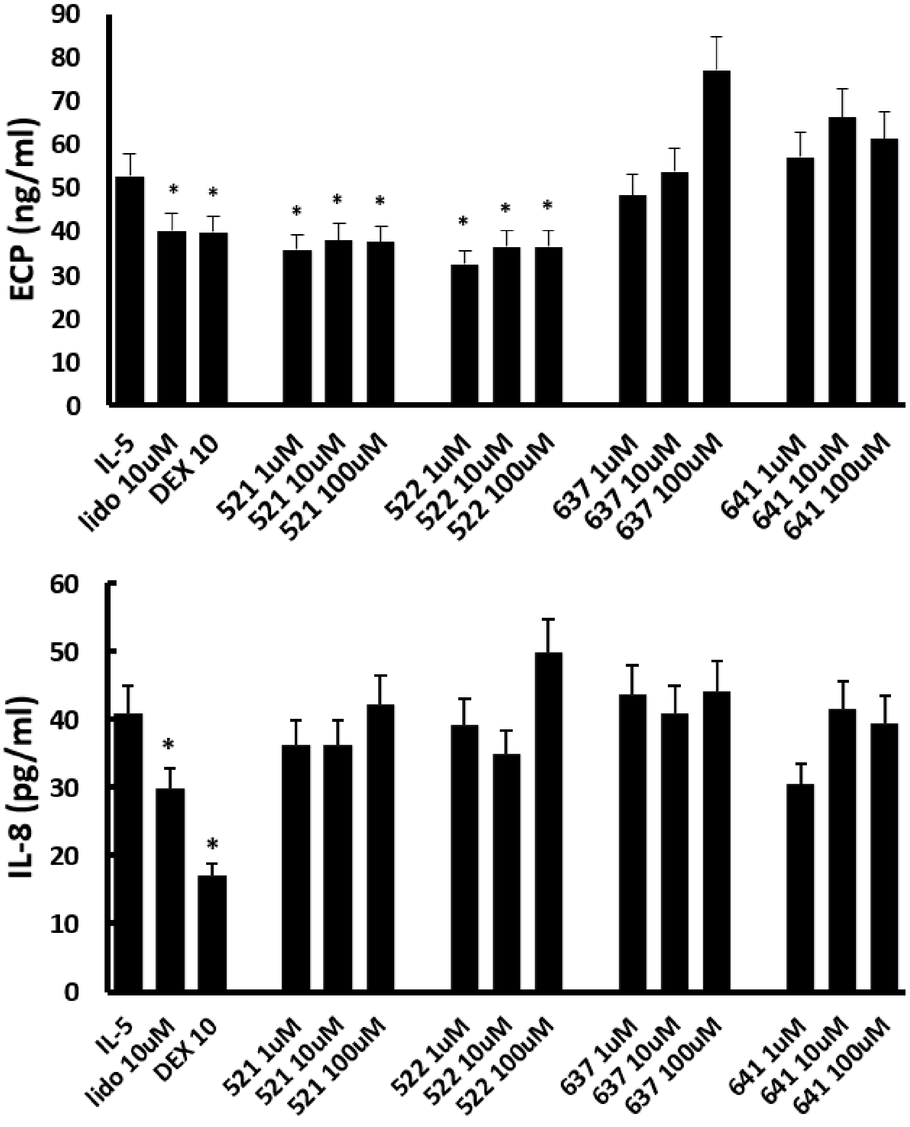

Figure S1. The effects of lidocaine-derived compounds EI521, E522, E637, and EI641 on IL-5-induced ECP and interleukin (IL)-8 production from eosinophils. IL-5-induced ECP production was significantly inhibited by compounds EI521 and EI522. IL-5-induced IL-8 production was not significantly inhibited by the compounds. lido, lidocaine; DEX, dexamethasone; * $p < 0.05$ compared with the IL-5 group, $n = 5$.

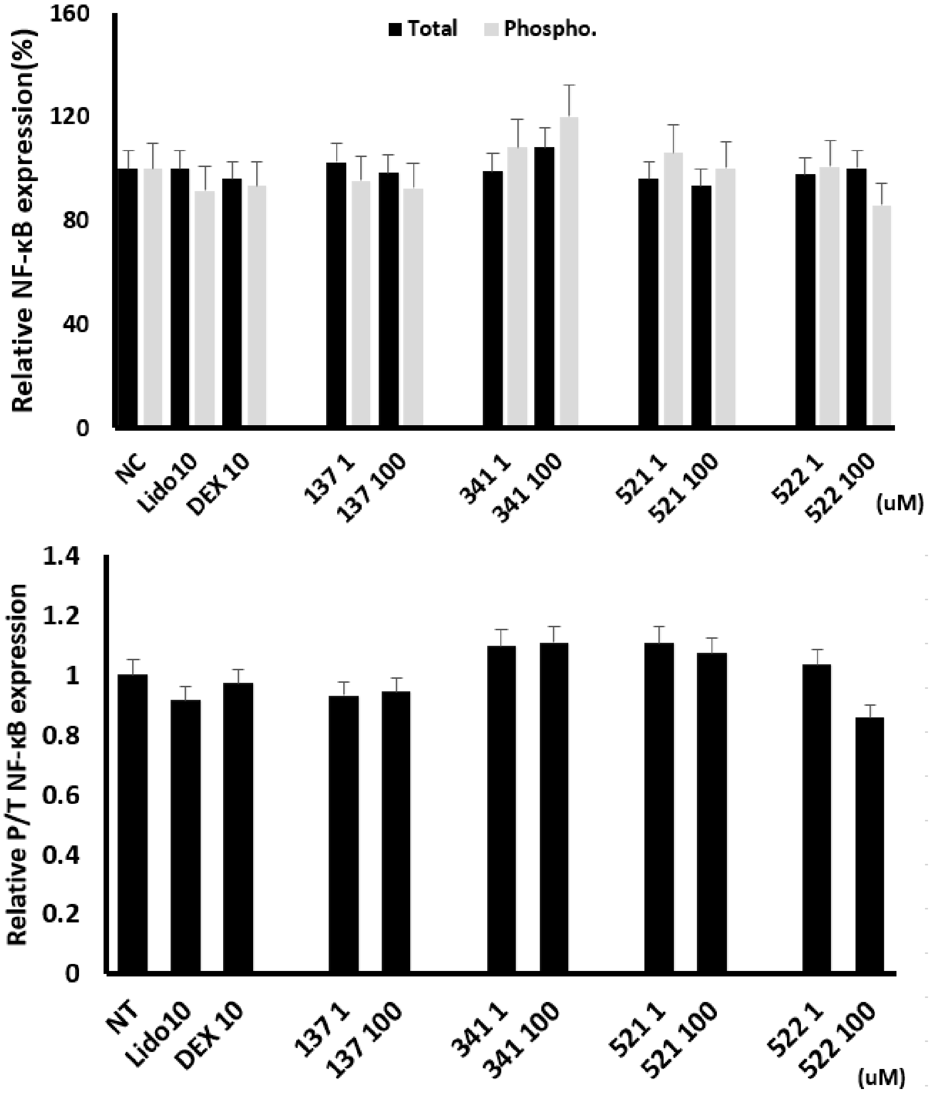

Figure S2. The effects of lidocaine analogs on IL-5-induced total and phosphorylated NF-κB expression in eosinophils. The compounds did not influence IL-5-induced phosphorylated NF-κB expression and relative phosphorylated/total NF-κB expression in eosinophils. NT, not-treated; Lido, lidocaine; DEX, dexamethasone, group, n = 7.

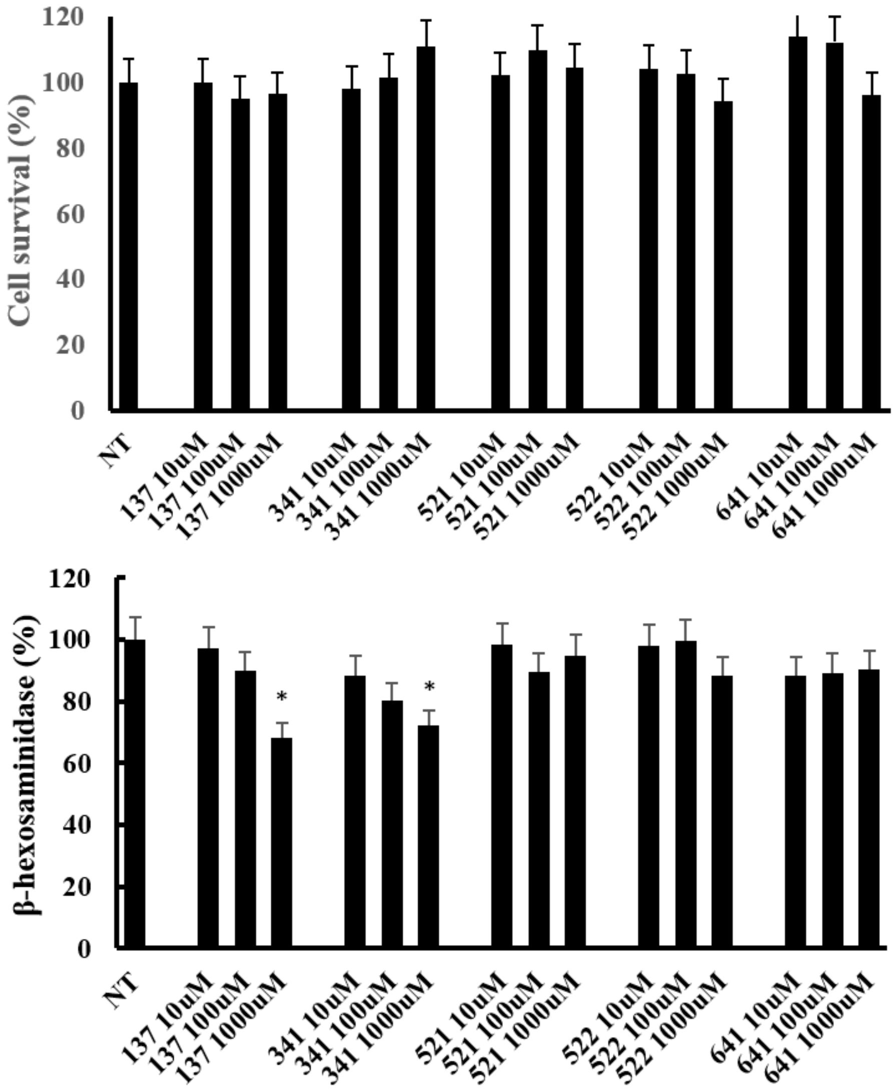

Figure S3: The effects of lidocaine-derived organic compounds on RBL-2H3 cell survival and degranulation. Organic compounds did not influence the survival of RBL-2H3 cells. At a dose of 1,000 μM, compounds 137 and 341 significantly inhibited the release of β-hexosaminidase from RBL-2H3 cells. NT, not-treated; * $p < 0.05$ compared with the NT group, n = 7.
